# Gradient Decomposition Methods for Training Neural Networks With Non-ideal Synaptic Devices

**DOI:** 10.3389/fnins.2021.749811

**Published:** 2021-11-22

**Authors:** Junyun Zhao, Siyuan Huang, Osama Yousuf, Yutong Gao, Brian D. Hoskins, Gina C. Adam

**Affiliations:** ^1^Department of Computer Science, George Washington University, Washington, DC, United States; ^2^Department of Electrical and Computer Engineering, George Washington University, Washington, DC, United States; ^3^Physical Measurement Laboratory, National Institute of Standards and Technology, Gaithersburg, MD, United States

**Keywords:** non-negative matrix factorization, gradient data decomposition, principal component analysis, memristor, non-idealities, ReRAM

## Abstract

While promising for high-capacity machine learning accelerators, memristor devices have non-idealities that prevent software-equivalent accuracies when used for online training. This work uses a combination of Mini-Batch Gradient Descent (MBGD) to average gradients, stochastic rounding to avoid vanishing weight updates, and decomposition methods to keep the memory overhead low during mini-batch training. Since the weight update has to be transferred to the memristor matrices efficiently, we also investigate the impact of reconstructing the gradient matrixes both internally (*rank-seq*) and externally (*rank-sum*) to the memristor array. Our results show that streaming batch principal component analysis (streaming batch PCA) and non-negative matrix factorization (NMF) decomposition algorithms can achieve near MBGD accuracy in a memristor-based multi-layer perceptron trained on the MNIST (Modified National Institute of Standards and Technology) database with only 3 to 10 ranks at significant memory savings. Moreover, NMF *rank-seq* outperforms streaming batch PCA *rank-seq* at low-ranks making it more suitable for hardware implementation in future memristor-based accelerators.

## Introduction

As artificial intelligence (AI) applications become ubiquitous in medical care, autonomous driving, robotics, and other fields, accuracy requirements and neural network complexity increase in tandem, requiring extensive hardware support for training. For example, GPT-3 is made up of ≈175 billion parameters and requires 285,000 central processing unit (CPU) cores and 10,000 graphics processing units (GPUs) to be trained on tens of billions of web pages and book texts (Langston, 2020). Moreover, the use of such significant computing resources has major financial and environmental impacts (Nugent and Molter, 2014; Strubell et al., 2020). New neuroinspired hardware alternatives are necessary for keeping up with increasing demands on complexity and energy efficiency.

Emerging non-volatile memory (NVM) technologies, such as oxygen vacancy-driven resistive switches, also known as ReRAM or memristors (Chang et al., 2011; Wong et al., 2012; Chen, 2020), can combine data processing and storage. Memristor matrices (crossbar arrays) use physical principles to enable efficient parallel multiply-accumulate (MAC) operations (Hu et al., 2018). This in-memory computing paradigm can achieve a substantial increase in speed and energy efficiency (Ceze et al., 2016) without the bottleneck caused by traditional complementary metal-oxide-semiconductor (CMOS) transistor-based von Neumann architectures. However, due to the inherent operational stochasticity of memristors in addition to manufacturing yield and reproducibility challenges, this emerging technology suffers from non-idealities. Thus, the accuracy of a neural network implemented with non-ideal memristor synaptic weights is not software-equivalent. To alleviate the undesirable effects of these devices, it is necessary to engineer better devices and improve the existing training algorithms.

This work investigates the use of Mini-Batch Gradient Descent (MBGD) for high accuracy training of neural networks with non-ideal memristor-based weights together with the use of gradient decomposition methods to alleviate the memory overhead due to the storage of gradient information between batch updates. An initial investigation (Gao et al., 2020) showed that the MBGD of moderate batch sizes (e.g., 128) can overcome the low accuracy of SGD for a one-hidden-layer perceptron network implemented with non-ideal synaptic weights trained on MNIST dataset. Accuracies of up to 86.5% were obtained for the batch sizes of 128 compared with only 50.9% for SGD. Although these results are promising, they are still far from the software equivalency of 96.5% at our studied network size.

**FIGURE 1:**
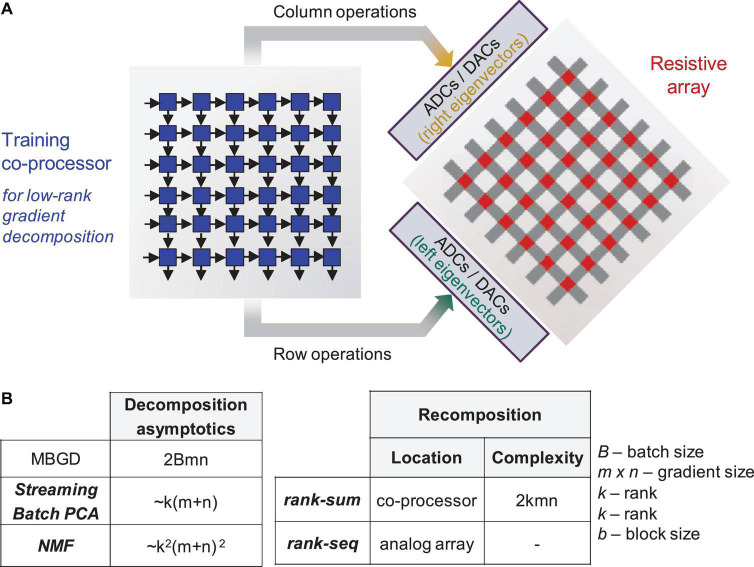
Training co-processor for decomposition. **(A)** Sketch showing an integrated system – a digital training co-processor will implement the best identified algorithm in an efficient way to support neural network training on non-ideal analog arrays. **(B)** Computational complexity for Mini-Batch Gradient Descent (MBGD), streaming batch principal component analysis (PCA), and non-negative matrix factorization (NMF), as well recomposition methods – rank summation (rank-sum) vs. rank-by-rank update (rank-seq). The respective eigenvectors are color coded.

Moreover, MBGD is memory intensive—particularly at higher batch sizes—since the gradient information needs to be stored before the batch update. We propose using a hardware co-processor to compress MBGD gradient data and work in tandem with the resistive array to support efficient array-level updates (Figure 1A). The first step toward this goal and the key question addressed by this paper is what decomposition algorithm should be mapped to a hardware co-processor to best support the training, particularly in neural networks implemented with non-ideal devices. Different common low-rank decomposition methods are available and have been extensively used in computer science literature to pre-process the dataset, remove noise and reduce the number of the network parameters (Garipov et al., 2016; Schein et al., 2016). Our prior work (Huang et al., 2020a,b) proposed streaming batch Principal Component Analysis (PCA) and showed that an accurate gradient matrix can be recomposed with as few as 3 to 10 ranks depending on the dataset complexity. Tests on CIFAR-10, CIFAR-100, and ImageNet showed near equivalent accuracy to MBGD at significant memory savings. However, in that work, non-ideal neural networks were not investigated.

In this study, we investigate the device-algorithm interaction which highlights the importance of hyperparameter optimization and stochastic rounding for overcoming the low-bit precision coding of the memristor weights. We propose an expansion of MBGD for larger batch sizes in conjunction with two gradient decomposition methods - Streaming Batch PCA and non-negative matrix factorization (NMF) - and recomposition methods based on rank summation (rank-sum) vs. rank-by-rank update (rank-seq) applied to a network with realistic memristor hardware models. For a m × n gradient matrix with batch size B, the MBGD cost is approximated at 2Bmn. By comparison, Streaming Batch PCA and NMF have asymptotic complexities of k(m+n) and k^2^(m+n)^2^, respectively (see Figure 1B). The issue of gradient recomposition in order to support weight updating is also investigated, considering that rank-sum would require additional overhead on the training co-processor, while for rank-seq it is possible to envision a series of rank-1 array level updates that support recomposition on the array itself. However, it is important to point out that these decomposition algorithms have high complexity requiring QR decompositions or iterative calculations when implemented at the algorithmic level and executed on a CPU. Dedicated hardware decomposers can be envisioned that support streaming operation on data flows.

The remainder of the paper is organized as follows. Section 2 has background information related to memristors and their applicability to neural networks, as well as an overview of decomposition algorithms. Section 3 describes the methodological details, the simulation environment, and the algorithms used. Section 4 introduces the evaluation of the proposed methodology on MNIST and its comparison with SGD and MBGD. Section 5 concludes with a discussion of the results.

## Related Work

### Resistive Switching Phenomena and Memristor Technology

The resistive switching phenomena was discovered in aluminum oxide in the early 1960s (Hickmott, 1962) and in other materials in the following decades (Argall, 1968; Dearnaley et al., 1970; Oxley, 1977; Pagnia and Sotnik, 1988). Due to the focus on silicon integrated circuits of the time, the technological potential of this phenomenon was not explored until the early 2000s, sparked by industry’s interest in the one transistor and one memristor (1T1R) cell for digital memories (Baek et al., 2004; Seo et al., 2004; Rohde et al., 2005).

These devices have a simple structure: the upper and lower layers are metal electrodes, and the middle layer is a dielectric layer, typically a transition metal oxide. The device behavior is driven by complex multi-physics phenomena, and it is not yet fully understood. However, the main model is based on the formation and reshaping of conductive filaments. When a voltage pulse is applied, the electronic and ionic conduction driven by local Joule heating causes the filament to reshape, thus changing the device resistance and programming the weight. When the voltage is removed, and the local Joule heating stops, the ions in the structure “freeze” in place, thus retaining the filament shape and its associated resistance/weight state providing memory to the system.

Due to the inherent stochastic nature of the ionic movement under Joule heating, the devices exhibit non-ideal characteristics, such as programming variability from cycle to cycle and from device to device, the asymmetry between the resistance increase (turn OFF – long term depression) and resistance decrease (turn ON – long term potentiation), read noise, and limited ON/OFF ratio or accessible resistance states. Other device indicators, such as device yield, read noise, and retention also impact their practical applicability (Gokmen and Vlasov, 2016; Lin et al., 2019).

### Memristor-Based Neural Network Training

A memristor crossbar can efficiently implement vector matrix multiplication using Ohm’s law for the input voltage to synaptic weight conductance multiplication and Kirchhoff’s law for the addition of the resulting currents. Together, these principles give rise to a vector dot product, which is the fundamental operation needed for fully-connected neural network layers (Prezioso et al., 2015). However, memristor non-idealities make the training process difficult (Adam et al., 2018). Therefore, the classification accuracies of *in-situ* training using non-volatile-memory hardware have generally been less than those of software-based training.

Several approaches have been used to mitigate these memristor device non-idealities. At the software level, binary neural networks (Chen et al., 2018) can use the devices as ON/OFF switches to reduce the impact of variability and conductance quantization. Alternatively, stochastic networks can exploit inherent cycle-to-cycle variability (Payvand et al., 2019; She et al., 2019). At the hardware level, more complex multi-memristor cells can be used (Boybat et al., 2018) to overcome asymmetry, limited bit precision and device variability at the expense of increased hardware overhead. Feedback circuitry can also be used to set the device to a well-defined value and mitigate the cycle-to-cycle variability of the devices (Serb et al., 2015). These solutions can be similarly applied to other types of emerging non-volatile memory technologies such as phase-change memory (Kim et al., 2019), magnetoresistive memory (Hirtzlin et al., 2019), ferroelectric-based memories (Berdan et al., 2020), among others.

A recent solution proposed by Ambrogio et al. (2018) has shown that batch analog systems can achieve equivalent training performance to that of the software but only at the costs of doubling the memory and exerting additional efforts in closed-loop training. Their proposed accelerator uses an analog short-term memory based on capacitors and transistors for fast and highly linear programming during training with only infrequent transfer to an analog long-term memory based on phase changes. The capacitive short-term memory is used to correct problems due to the imperfections in programming long-term phase change memories (Haensch et al., 2018). This approach, which combines the advantages of two device technologies, is feasible. However, it relies on duplicate short-term and long-term memories. Additionally, any imperfections of the short-term memory also need to be managed in hardware. A working prototype has not yet been demonstrated. Nevertheless, understanding how to leverage alternative algorithms and architectures is critical since evidence suggests that certain algorithms, like batch update, are more resilient to the non-idealities of various devices (Kataeva et al., 2015; Gao et al., 2020; Gokmen and Haensch, 2020).

### Matrix Decomposition Algorithms

Rather than using a duplicative short-term memory, linear algebra techniques can be used to compress gradient data and support efficient array-level updates. Principal component analysis (PCA), a commonly used decomposition method, projects high-dimensional data into low-dimensional subspaces. Through computing and analyzing the underlying eigenspectrum, the variance in the data is maximized. Streaming PCA (Oja, 1982), streaming history PCA (Burrello et al., 2019; Hoskins et al., 2019), and streaming batch PCA (Huang et al., 2020b) were all developed based on the core PCA algorithm. Streaming batch PCA can extract an approximation of a full matrix from samples of its contributed parts by combining bi-iterative stochastic power iterations (Vogels et al., 2019) with QR factorization to produce low rank approximations of stochastic rectangular matrices. This method reduces gradient storage and processing requirements brought by MBGD and is composed of a batch of randomly generated rank-1 matrices of forward propagated activations and backpropagated errors.

However, streaming batch PCA has no restriction on the sign of the data element, so negative values can appear in the matrix factorization. Even if all the values are strictly positive, such as in an image, the decomposition may include negative terms. This oscillatory behavior, while usually harmless, causes challenges when computation is done at the physical level: for instance, summation on memristor devices which are not inherently reversible in their programming behavior. By contrast, the Non-Negative Matrix Factorization (NMF) algorithm (Paatero and Tapper, 1994; Wang et al., 2015) calculates the decomposition by adding the non-negative constraints which results in additive features.

The NMF decomposition is particularly meaningful when the gradient information is mapped on a memristor matrix for physical recomposition. NMF can decrease the overlap between ranks, eliminating the oscillatory behavior during summation that exists in a standard PCA decomposition. This is crucial for devices that do not have a linear and symmetric weight update.

The streaming batch PCA algorithm and NMF decomposition algorithms will be used in the following sections to approximate the MBGD gradient and train a fully connected network to classify MNIST handwritten digits with high accuracy, despite device non-idealities.

## Method Details

### Streaming Batch Principal Component Analysis

Streaming batch PCA or SBPCA (Huang et al., 2020b) is used to decompose the gradient information from MBGD. It compresses batch data in the neural network training period through rank-*k* outer product updates. The streaming batch PCA can expedite gradient descent training and decrease the memory cost by generating a stochastic low-rank approximation of the gradient. Gradient descent reduces the error between the predicted value of the neural network and the actual value by updating the parameters to minimize the result of the loss function,


$$\Theta_{p}=\Theta_{p}-\alpha *\nabla_{\Theta }l,$$

where Θ_*p*_ is the weight matrix of layer *p*, α is learning rate, *l*(Θ) is the loss function, and $\nabla_{\Theta }l=\frac{\partial l\,\left(\Theta_{p}\right)}{\partial \Theta_{p}}$ is the gradient.

To extract significant batch gradient data, average out the noise due to non-ideal memristor weights, and improve the network accuracy, a streaming low-rank approximation of ${\hat{\nabla }}_{\Theta }^{\left(k,B\right)}\,l$ is obtained by the Streaming Batch PCA. The gradient is approximated for a batch of size *B* and the top-*k* most important *k* ranks as follows:


$${\hat{\nabla }}_{\Theta }^{\left(k,B\right)}\,l=\hat{X}\cdot \hat{\Sigma }\cdot {\hat{△}}^{T},$$

where $\hat{X}\in {\mathbb{R}}^{n\times k}$ and $\hat{△}\in {\mathbb{R}}^{n\times k}$ denote the left singular matrix and right singular matrix, respectively. $\hat{\Sigma }=d\,i\,a\,g\,\left(\vec{\sigma }\right)\in {\mathbb{R}}^{k\times k}$ is a diagonal matrix, which has on its diagonal the corresponding singular values $\vec{\sigma }$ for the top *k* ranks. In the Streaming Batch PCA algorithm, the input $\vec{x}\in {\mathbb{R}}^{1\times m}$ and the error $\vec{\delta }\in {\mathbb{R}}^{1\times n}$ help to update $\hat{X}$ and $\hat{△}$. Based on Oja’s rule and stochastic power iterations (Oja, 1992; Huang et al., 2020a), $\hat{X}$ and $\hat{△}$ are updated separately and bi-iteratively in a streaming fashion with an averaged block size *b < B*, followed by re-orthogonalization via QR factorization. Our QR factorization is defined to have non-increasing values on the diagonal of the *R* matrix. For updating $\hat{X}$, we use


$$\hat{X}\leftarrow Q\,R\,\left[\frac{i}{i+1}\cdot \hat{X}+\frac{1}{i+1}\cdot \frac{{\hat{x}}^{T}\,\hat{\delta }\,\hat{△}\,{\hat{\Sigma }}^{-1}}{b}\right],$$

where $\frac{i}{i+1}$ represents the convergence coefficient and $\hat{X}$ decays with each QR factorization, running from *i = 1* until reaching *i = B/b*. The update of $\hat{\Sigma }$ is similar,


$$\hat{\Sigma }\leftarrow \frac{i}{i+1}\cdot \hat{\Sigma }+\frac{1}{\left(i+1\right)}\,\sum_{\text{rows}}\frac{\left(\hat{\text{x}}\,\hat{\text{X}}\right)\,⊙\left(\hat{\delta }\,\hat{△}\right)}{b},$$

where ⊙ is the Hadamard (elementwise) matrix product.

From the standpoint of computational complexity, Streaming Batch PCA with k-ranks requires *4Bk(m+n)+$\frac{B}{b}\cdot$4k(m+n)* floating point operations (FLOPs) where $\frac{B}{b}\cdot$4k(m+n) is for the batch size (B) / block size (b) times QR factorizations. Overall, the complexity tends to scale as k(m+n), leading to an overall reduced computational load as compared to MBGD. However, the recomposition complexity scales as kmn, What this means is that recreating the approximation of the gradient is more computationally expensive than getting the most important eigenvectors making the recomposition calculation the most expensive part the algorithm.

### Non-negative Matrix Factorization

The Non-Negative Matrix Factorization (NMF) (Lee and Seung, 1999) algorithm decomposes a non-negative matrix into two non-negative left and right matrices $\hat{X}\in {\mathbb{R}}_{+}^{m\times k}$ and $\hat{△}\in {\mathbb{R}}_{+}^{k\times n},$ respectively.

However, the gradient ∇_Θ_*l* is not non-negative. This is why in our NMF algorithm, we first start with a batch size *B* approximation of ∇_Θ_*l*, and then use the rectified linear unit (ReLU) activation function to restrict the sign of gradient ∇_Θ_*l* by its unilateral inhibition feature, whereby ReLU(*v*) = *max*(*v*, 0). The goal is to approximate the positive and negative parts separately with two sets of *k-*rank matrices such that $\hat{\nabla }\,l_{P}={\hat{X}}_{P}\cdot {\hat{△}}_{P}$ and $\hat{\nabla }\,l_{N}={\hat{X}}_{N}\cdot {\hat{△}}_{N}$. Four random matrices ${\hat{X}}_{P},{\hat{X}}_{N}\in {\mathbb{R}}_{+}^{m\times k}$ and ${\hat{△}}_{P},{\hat{△}}_{N}\in {\mathbb{R}}_{+}^{n\times k}$ are randomly initialized from a Gaussian distribution at the beginning of training with a standard deviation calculated from the root of the mean values of the gradient over the rank *k*, $\sqrt{\frac{\nabla_{\Theta }{\bar{l}}_{P}}{k}}$ and $\sqrt{\frac{\nabla_{\Theta }{\bar{l}}_{N}}{k}}$. Then, we use a modified version of the Fast HALS (Hierarchical Alternating Least Squares) (Cichocki and Phan, 2009) algorithm to alternately update the left and right matrices. To do the minimization, we assume a pair of loss functions of the form $\frac{1}{2}\,{||\nabla_{\Theta }l_{P}^{\left(k\right)}-{\hat{X}}_{P\,k}\,{\hat{△}}_{P\,k}^{T}||}_{F}^{2}$, where *k* is the rank and *F* is the Frobenius norm, with one loss function for the positive matrix and a similar one for the negative matrix. This product of the left (${\hat{X}}_{P\,k}$) and right (${\hat{△}}_{P\,k}^{T})$ matrices best approximates the non-negative gradient when these loss functions are minimized. During the non-negative part iteration update, the quantities ${\hat{X}}_{P}^{T}\,{\hat{X}}_{P}$ and ${\hat{△}}_{P}\,{\hat{△}}_{P}^{T}$ are calculated. The diagonal matrices $D_{X}\leftarrow \mathrm{Diag}\,{\left({\hat{X}}_{P}^{T}\,{\hat{X}}_{P}\right)}^{-1}$ and $D_{△}\leftarrow \mathrm{Diag}\,{\left({\hat{△}}_{P}\,{\hat{△}}_{P}^{T}\right)}^{-1}$ are calculated to scale the updates (Cichocki et al., 2009). Similar quantities are calculated for ${\hat{X}}_{N}$ and ${\hat{△}}_{N}$. With this basic framework in mind, we can iteratively, for as many as *P* cycles, update the positive (or negative) decomposition by

${\hat{X}}_{P}\leftarrow \text{ReLU}\,\left({\hat{X}}_{P}+\left(-{\hat{X}}_{P}\,{\hat{△}}_{P}\,{\hat{△}}_{P}^{T}+\nabla_{\Theta }{\bar{l}}_{P}\,{\hat{△}}_{P\,k}^{T}\right)\,D_{△}\right)$, and


$${\hat{△}}_{P}\leftarrow \text{ReLU}+\left({\hat{△}}_{P}+\left(-{\hat{△}}_{P}{\hat{X}}_{P}^{T}{\hat{X}}_{P}+\nabla_{\Theta }{\bar{l}}_{P}{\hat{X}}_{P\,k}\right)D_{\text{X}}\right)).$$

The number of iterations, *P*, will depend on the desired level of convergence as well as the initialization. The number of iterations can be reduced by streaming the current best estimates for ${\hat{X}}_{P},{\hat{△}}_{P}$ and ${\hat{X}}_{N},{\hat{△}}_{N}$ from batch to batch after the first random initialization, as we do in our case. In this work, we explored using a fixed, 200 iterations to understand the impact of NMF factorization on training. We also studied doing these operations with one iteration to see how streaming would impact training, see Section 3 and Supplementary Figure 2.

After convergence, the new left gradient matrix $\hat{\nabla }\,l_{P}={\hat{X}}_{P}\cdot {\hat{△}}_{P}$ and right matrix $\hat{\nabla }\,l_{N}={\hat{X}}_{N}\cdot {\hat{△}}_{N}$ would be generated. At the end, the low-rank matrix approximation is ${\hat{\nabla }}_{\Theta }^{\left(k,B\right)}\,l=\hat{\nabla }\,l_{P}-\hat{\nabla }\,l_{N}$. It is important to understand that, while this method produces a potentially optimal and non-oscillating decomposition, it still relies on summing and reconstructing the batch gradient. This makes it much more computationally complex than the Streaming Batch PCA algorithm. However, its memory overhead could be improved and its hardware mapping will be explored in the future. For this work, we are primarily interested in the impact of the decomposition on training.

Assuming the sequential least squares minimization (e.g., HALS) is done in *p* iterations, the FLOPs required for NMF scales with $3mn+2mk+2nk+2mnk\left(n-1\right)+2p(k^{2}\left({\left(m+n\right)}^{2}-m-n\right)+mnk\left(m+n-4\right)+4k\left(m+n+\frac{1}{2}\right)$. The $\left(k^{2}\,\left({\left(m+n\right)}^{2}-m-n\right)+m\,n\,k\,\left(m+n-4\right)+4\,k\,\left(m+n+\frac{1}{2}\right)\right)$ calculations are for the ${\hat{X}}_{P}$ and ${\hat{△}}_{P}$ or ${\hat{X}}_{N}$ and ${\hat{△}}_{N}$ updates in one iteration. As noted previously, the overall computational complexity scales as k^2^(m+n)^2^ making it at this time more computationally complex than MBGD. However, should this performance be improved, it would be very advantageous since the NMF algorithm has a better performance when training networks rank-by-rank, or using the *rank-seq* operation as discussed below.

### Rank Gradient Recomposition Methods

**FIGURE 2:**
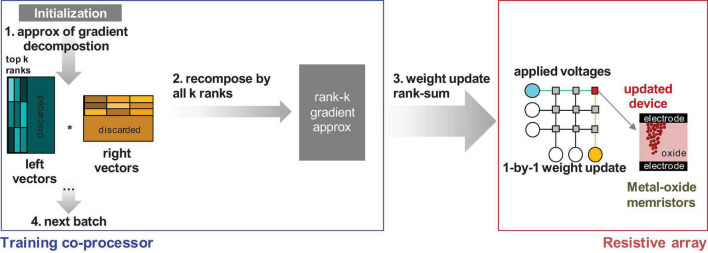
Transfer principle of the gradient approximation information to the memristor array for the rank summation outside the array (rank-sum). The training co-processor would have to support rank-k gradient recomposition, thus increasing the hardware overhead.

The contrast between the oscillatory behavior of the streaming batch PCA and the additivity of the NMF decomposition methods becomes significant when considering the memristor weight updates in hardware. How these updates are performed is important for understanding the choice of algorithm on performance. One option is to do gradient summation across the ranks of interest outside the analog memory crossbar array before transfer. During training, individual samples are used to update the compressed *k*-rank representation of the gradient ${\hat{\nabla }}_{\Theta }^{\left(k,B\right)}l$ based on the calculated $\hat{X}$, $\hat{\Sigma }$, and $\hat{△}$. At the end of a training batch, the gradient is recomposed and then added to the matrix in total ${\hat{\nabla }}_{\Theta }^{\left(k,B\right)}\,l=\hat{X}\cdot \hat{\Sigma }\cdot {\hat{△}}^{T}$ by sequentially updating each weight one by one. We call this approach the *rank-sum* update and summarize it in Figure 2.

**FIGURE 3:**
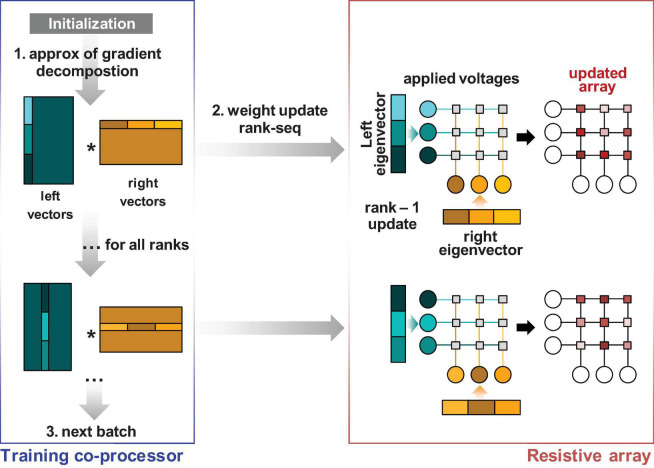
Transfer principle of the gradient approximation information to the memristor array via rank-by-rank (rank-seq) transfer. The gradient recomposition can be done by physical summation of rank-1 updates at the array level, thus reducing the hardware overhead for the training co-processor.

However, *rank-sum* is inefficient since (a) the data must be multiplied out and summed on the array and (b) the data must be transferred one by one into each of the individual memristor devices. The estimated computational complexity of this operation, as noted in Figure 1, is 2kmn. A more efficient implementation for pipelining requires the gradient summation inside the array using the update properties of the memristor devices. After producing an approximation of the gradient, the weight matrix is updated rank by rank, and the gradient is summed on the memory devices using outer product update operations. Outer product operations can be done in multiple ways, either using pulses on the rows and columns (Kataeva et al., 2015; Gokmen and Vlasov, 2016) or by relying on an exponential dependence on the applied bias on the rows and columns to multiply out the gradient (Kataeva et al., 2015). Outer product operations restrict the updates, because of the limited row/column access, to rank-1 updates. Consequently, ${\hat{\nabla }}_{\Theta }^{\left(j,B\right)}\,l$ is a rank-1 matrix for the *j*th rank from the matrix product for the column *j* in $\hat{X}$, $\hat{\Sigma }$, and Δ^:∇^Θ(j,B)⁢l=X^m,j⁢Σ^j⁢Δ^Tn,j. The column number is less than or equal to rank *k*. Unlike the *rank-sum* method, the *rank-seq* method does not pre-sum ${\hat{\nabla }}_{\Theta }^{\left(k,B\right)}\,l$ for all the ranks *k*. The matrix ${\hat{\nabla }}_{\Theta }^{\left(j,B\right)}\,l$ is used to calculate the necessary updates for rank *j* to be transferred to the memristor matrix where the gradient is recomposed at the physical level. We call this method the *rank-seq* update and show its principles in Figure 3.

It is worth pointing out that in a traditional floating-point software implementation, the two algorithms are equivalent within rounding error. However, when the gradient information needs to be transferred to a non-ideal memristor circuit, the two methods differ. *Rank-sum* updates the gradient information to the memristor crossbar only once, while the *rank-seq* needs *k* updates for *k* ranks. Updates to non-ideal memristors are accompanied by a loss in gradient precision, which is the reason that *rank-seq* to be expected to have lower accuracy than *rank-sum* for non-overlapping ranks. However, *rank-seq* is more efficient since it requires less digital computation and hardware overhead.

### Stochastic Rounding

As part of the gradient transfer, the accuracy of the quantization of the weight update is also investigated in relation to the device properties. Although in theory the memristor has analog programmability to any desired state between the ON and the OFF, the device in practice has low bit precision. The reason for low bit precision is that each state can naturally decay and can be impacted by reading disturbs or be impacted by the programming of neighboring devices (Lin et al., 2019). Therefore, the number of conductance levels reliably accessible and distinguished from each other is limited. This quantization of the weight update introduces errors due to the lower bit precision. Since the memristor conductance change is related to the number of applied pulses (an integer), the respective weight modification needs to be rounded appropriately to a lower bit precision. Rounding-to-nearest is the method commonly used (Chen et al., 2017). However, it seems to cause a premature conversion to sub-optimal accuracies at higher batch sizes due to small gradients and low bit precision causing delta weight approximation to zero.

In this work, stochastic rounding is investigated instead to overcome this quantization error vanishing gradient issue in limited precision weights. Stochastic rounding, proposed in the 1950s and 1960s (Forsythe, 1950; Barnes et al., 1951; Hull and Swenson, 1966), can be particularly useful in deep network training with low bit precision arithmetic (Gupta et al., 2015). A real value *r* which lies between floor value (*r*_1_) and ceiling value (*r*_2_) is stochastically rounded up to *r*_2_ with probability (*r*-*r*_1_)/(*r*_2_-*r*_1_) and down to *r*_1_ with probability (*r*_2_-*r*)/(*r*_2_-*r*_1_). The average error of this rounding method is zero, since the expected value of the result of stochastically rounding *r* is *r* itself. Using this stochastic rounding method, some of the sub-bit information that is discarded by a deterministic rounding scheme can be maintained.

## Results

### Network Structure and Simulation Environment

A multi-layer perceptron to be trained on the MNIST dataset is chosen. It has high software accuracies and weight matrices map directly to memristor crossbars, making it suitable for exploring device-algorithm interactions. The impact of the proposed methods can be quantified without any interfering effects from a training optimizer, potentially unoptimized deep network or an overly challenging dataset. The network structure is 400 (input layer) - 100 (hidden layer) - 10 (output layer). The hardware mapping and training on the MNIST dataset is available in NeuroSim V3.0. NeuroSim V3.0 is an open-source integrated simulation framework based on C++ for benchmarking synaptic devices and array architectures via system-level learning accuracy and hardware performance indicators (Chen et al., 2017). As part of this work, modules for MBGD, streaming batch PCA and NMF as well as two weight transfer methods: *rank-sum* and *rank-seq* were implemented and integrated with the existing NeuroSim V3.0 capabilities.

**FIGURE 4:**
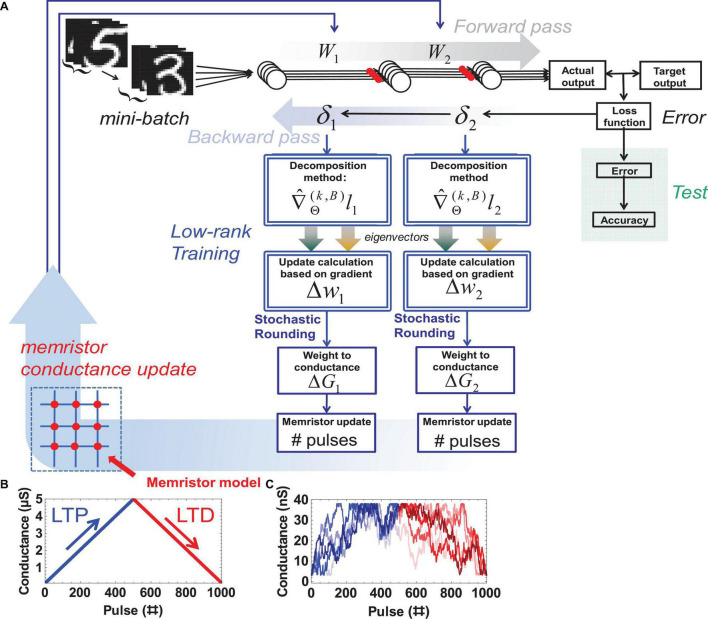
The code structure and device models **(A)** expanded NeuroSim v3.0 with added modules and functions highlighted with a double border. **(B)** Ideal memristor with linear symmetric and reproducible weight update and a large ON / OFF ratio vs. **(C)** Non-ideal memristor model with smaller ON / OFF ratio, weight update nonlinearity, and variability (5 cycles shown).

The algorithmic flow between the modules and the device models used are shown in Figure 4.

The gradient information obtained during backpropagation is decomposed according to the desired method. The desired weight update is calculated in the form of pulses to update the conductance in hardware. This paper uses the ideal device model and the non-ideal (real) device model with the 1T1R configuration of NeuroSim V3.0 to avoid leakage effects. The ideal device model assumes a reproducible linear relationship between the applied number of pulses and the obtained conductance (Figure 4B). In the non-ideal device model, there is non-linearity between the applied pulses and the conductance, which leads to imperfect weight programming and variability in the operation. The nonlinearity values for long term potentiation (LTP) and long term depression (LTD) are 2.40 and −4.88, respectively. The cycle-to-cycle variation is 3.5%. This stochasticity is sufficiently large that sending an “increase weight” pulse can even randomly lead to a “decreased weight” and vice versa (Figure 4C). Other hardware parameters are the default values of NeuroSim, for example, the read noise is 0, and the minimum and maximum conductance are ∼3nS and 38 nS, respectively. These default values are extracted from fitting experimental weight update data derived from Ag:a-Si devices (Jo et al., 2010; Chen et al., 2017). For this work, a device with 500 levels is assumed (approximately 9-bit precision). Each change in level is assumed to correspond to one update pulse, with 500 pulses ultimately putting the device in the fully OFF or fully ON state.

### Rounding Effects of the Weight Update

**FIGURE 5:**
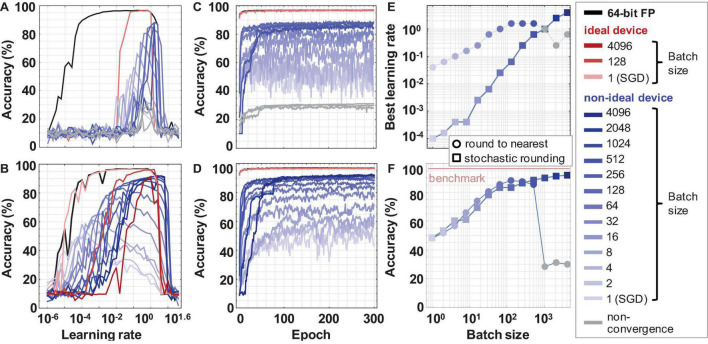
The MBGD results for rounding to nearest and stochastic rounding. Learning rate windows for ideal and non-ideal device models and batch sizes using **(A)** rounding-to-nearest vs. **(B)** stochastic rounding of the delta weight. Best convergence curves for the optimal learning rate for each batch size using **(C)** rounding-to-nearest vs. **(D)** stochastic rounding. **(E)** Optimal learning rate corresponding to the highest accuracy vs. the batch size for non-ideal devices. It is observed that the learning rate increases supra-linearly with the batch size. **(F)** Best accuracy at the optimal learning rate vs. the batch size. In **(E)** and **(F)**, the colorful plot markers represent results at convergence. The faded gray markers for the rounding-to-nearest case represent the inability of the network to train properly due to vanishing gradients in this low precision model. The values for the batch sizes 1 to 4096 are obtained using the realistic memristor model. Representative results for software and ideal device model are also included for comparison.

Figure 5 shows the training on the MLP network with software (64-bit floating-point precision), ideal memristor device (500 levels, 9-bit) and real device model (500 levels, 9-bit with cycle-to-cycle variability and non-linearity). The MNIST testing accuracies in the regular round-to-nearest truncation vs. the stochastic truncation is determined across various batch sizes in a logarithmic search of the learning rate domain. It can be observed that a network implemented with limited precision memristor devices, but no other non-idealities, achieves SGD accuracy 96.5% similar to a traditional software floating-point implementation. However, the quantization of the weight update shrinks the learning rate window dramatically. Whereas the floating-point implementation can achieve an accuracy > 95% for any learning rate between 0.001 and 1, the low precision memristor-based network can only train with a learning rate between 0.1 and 1 (Figure 5A). When stochastic rounding is used, the learning rate window for the quantized memristor model widens significantly, resembling the floating-point implementation (Figure 5B). This result highlights the importance of hyperparameter optimization and hardware-sensitive rounding in these low-precision networks.

Learning rate optimization was used to obtain these best accuracy results. The convergence curves for different device models, different batch sizes, and the two rounding methods were run for learning rates spanning eight orders of magnitude from 10^–6^ (0.000001) to 10^1.6^ (≈ 40). To optimize the search, this range was explored in logarithmic steps. The learning rates corresponding to the best accuracy for each test set are plotted in Figure 5E. As the batch size increased, the value of the optimal learning rate also increased. The learning rates for the round-to-nearest method are higher than the stochastic rounding method, despite their accuracies being similar. This might be due to the fact that stochastic rounding applied to these limited-bit precision systems can still, over many operations in the time series, on average, keep track of some sub-bit information. The stochastic rounding applied across the weights in the array can preserve statistically more gradient information and carry it over to the next back propagation iterations (Gupta et al., 2015). By comparison, the round-to-nearest truncation discards such gradient information.

Overall, it can be observed that the accuracy increases almost linearly with the log of the batch size for medium batch sizes (up to 128) for both round-to-nearest and stochastic rounding (Figure 5F). It plateaus at higher batch sizes converging to the MBGD floating-point software accuracy for higher batch sizes (Table 1). For our implementation, the MBGD at large batch sizes are similarly needed to overcome the gradient noise due to the non-ideal memristor synaptic weights. These results show that ideal memristor behavior, while desirable, is not needed on a single layer perceptron. The effects are likely to be even more apparent in larger fixed precision networks due to compounding effects as seen by related work (Gupta et al., 2015). Existing memristors can be used successfully despite their non-idealities and neural networks implemented with real memristor models can achieve software equivalency using appropriate algorithmic methods for training

**TABLE 1:** Best accuracy observed between the rounding methods at different batch sizes.

		MBGD Rounding-to-nearest		MBGD Stochastic Rounding	
Synaptic weight	B	Best LR	Best Accuracy (%)	Best LR	Best Accuracy (%)
64-FP (benchmark)	1	10^–0.8^ = 0.1585	96.81	Same	
Ideal device	1	10^–0.2^ = 0.6310	96.53	10^–0.4^ = 0.3981	96.5
Real device	1	10^–1.4^ = 0.0398	48.17	10^–4^ = 0.0001	48.45
	2	10^–1.2^ = 0.0631	51.71	10^–3.8^ = 0.0002	53.34
	4	10^–0.1^ = 0.1000	56.68	10^–3.4^ = 0.0004	60.28
	8	10^–0.8^ = 0.1585	65.88	10^–3.4^ = 0.0004	61.13
	16	10^–0.6^ = 0.2512	74.02	10^–2.6^ = 0.0025	69.34
	32	10^–0.2^ = 0.6310	80.58	10^–2.2^ = 0.0063	78.24
	64	10^1^ = 1.0000	85.50	10^–1.6^ = 0.0251	83.35
	128	10^0.2^ = 1.5849	88.24	10^–1.2^ = 0.0631	86.49
	256	10^0.2^ = 1.5849	87.48	10^0.2^ = 0.2512	88.53
	512	10^0.2^ = 1.5849	85.43	10^–0.2^ = 0.6310	90.06
	1024	10^0^ = 1.0000	28.38	10^0^ = 1.0000	91.28
	2048	10^–0.6^ = 0.2512	31.20	10^0.4^ = 2.5119	91.99
	4096	10^–0.2^ = 0.6310	29.99	10^0.6^ = 3.9811	91.93
64-FP	4096	10^0.2^ = 1.5849	93.42	Same	
	8192	10^0.2^ = 1.5849	90.73	Same	

### Streaming Batch PCA With Ideal vs. Non-ideal Weights

An in-depth investigation was done to explore how the accuracy changes with the rank, batch size and transfer method, and the difference between streaming batch PCA algorithm and full rank MBGD. Two batch sizes were investigated: 128 and 4096. The learning rates used for this streaming batch PCA investigation correspond to the best accuracies obtained by these batch sizes for MBGD. The decomposition method was applied to both layers at the same rank. The ranks investigated were 1, 3, and 10.

**FIGURE 6:**
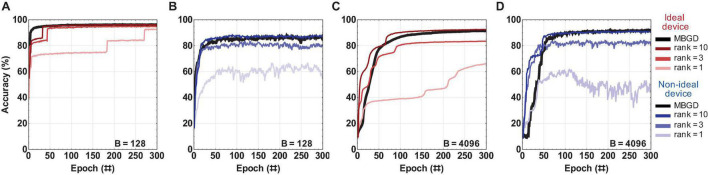
The rank-sum streaming batch PCA results for a multi-layer perceptron with ideal vs. non-ideal memristive weights at different ranks and batch sizes. **(A,B)** Convergence curves using the rank-sum weight transfer method at batch size = 128; and similarly **(C,D)** at batch size = 4096. The results show that a low-rank gradient decomposition can approximate the MBGD results fairly well, particularly for ranks 3 and 10. For all these experiments, the rounding method is stochastic and block size = 32.

Figure 6 summarizes the *rank-sum* results and as expected, the accuracy of the rank 1 results was lower than that of rank 3 and rank 10 for both batch size 128 and 4096 for both the device models. The performance for the ideal device model shows that the performance slightly decrease for MBGD at high batch size. However, the performance for non-ideal devices increases with the batch size. The rank-3 decomposition does seem to perform well by comparison with MBGD, particularly at the larger batch size. The convergence performance of rank 10 is at the same level as that of MBGD for the *rank-sum* transfer method. Additionally, with the increase in the rank, the convergence curve tends to smoothen and converge somewhat faster, achieving the desired accuracy in ≈ 25 epochs. The investigation of the impact of block size *b* is included as Supplementary Figure 1.

**FIGURE 7:**
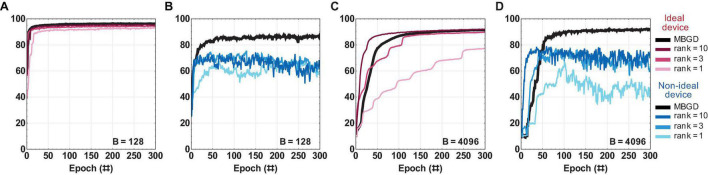
The rank-seq streaming batch PCA results for a multi-layer perceptron with ideal vs. non-ideal memristive weights at different ranks and batch sizes. **(A,B)** Convergence curves using the rank-seq weight transfer method at batch size = 128; and similarly **(C,D)** at batch size = 4096. The results show that the streaming batch PCA using rank-seq transfer method cannot approximate the MBGD results, even at high ranks. For all these experiments, the rounding method is stochastic and block size = 32.

While the *rank-sum* weight transfer method works very well for streaming batch PCA and achieves close to MBGD performance, its full hardware implementation would be difficult since the gradient approximation needs to be recomposed externally prior to being transferred to the memristor matrix. By contrast, gradient recomposition of *rank-seq* requires minimal hardware overhead. The results for *rank-seq* are summarized in Figure 7.

For ideal device, the accuracies are similar for both rank-sum and rank-seq. By comparison, for the non-ideal devices, accuracies around ≈ 70% are obtained for ranks 3 and 10 at both batch sizes 128 and 4096. This is 15 to 20 percentage points lower than the *rank-sum* and full rank MBGD results. These results show that the streaming batch PCA using *rank-seq* transfer method cannot approximate the MBGD results, even at high ranks. This is likely because the principal components of streaming batch PCA can have positive and negative elements, creating an oscillatory effect due to the programming of the non-ideal memristive weights (Scholz et al., 2008). This effect is observed indirectly in the noisy convergence curves.

### Streaming Batch PCA With Ideal vs. Non-ideal Weights

**FIGURE 8:**
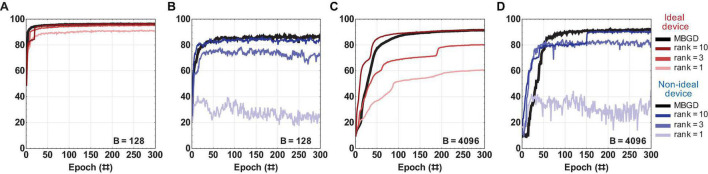
The rank-sum Non-Negative Matrix Factorization (NMF) results for a multi-layer perceptron with ideal vs. non-ideal memristive weights at different ranks and batch sizes. **(A,B)** Convergence curves using the rank-sum weight transfer method at batch size = 128; and similarly **(C,D)** at batch size = 4096. The results show that the NMF can approximate fairly well the MBGD results, particularly for ranks 3 and 10. For all these experiments, the rounding method is stochastic.

Figure 8 summarizes the *rank-sum* NMF results for the different ranks at the two different batch sizes and compares them with full rank MBGD. For ideal device, the rank 1 has lower performance, but rank 3 and rank 10 can approximate MBGD well, particularly at batch size 128. For non-ideal devices, the NMF can approximate the gradient information fairly well, particularly at rank 10. Rank 1 has extremely poor performance, similar to SGD. Rank 3 performs well and can converge, but its accuracy is still ≈ 5% to 10% lower than the equivalent MBGD result at the respective batch size. It is also worth noting that a decline in the accuracies of these lower ranks can be observed as the training progresses. Higher rank is needed to observe satisfactory accuracy and training stability. The result of rank 10 was only 1% to 2% lower than that of the MBGD algorithm. One reason for the high accuracy in the case of rank 10 is that because the second layer has only 10 neurons, rank 10 is actually equivalent to full rank training in the last layer, though not in the first layer.

**FIGURE 9:**
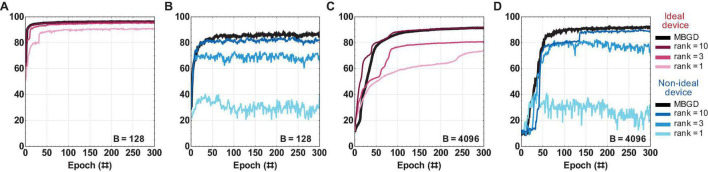
The rank-seq NMF results for a multi-layer perceptron with ideal vs. non-ideal memristive weights at different ranks and batch sizes. **(A,B)** Convergence curves using the rank-seq weight transfer method at batch size = 128; and similarly **(C,D)** at batch size = 4096. The results show that the NMF using rank-seq transfer method can approximate the MBGD results well at high ranks. For all these experiments, the rounding method is stochastic.

The results for the *rank-seq* transfer method applied to NMF are shown in Figure 9. For the ideal device, the accuracies are similar to rank-sum as expected. By comparison, for the non-ideal devices, rank 3 achieves ≈ 70% accuracy at batch size 128 and ≈ 80% accuracy at batch size 4096. For rank 10, the NMF algorithm performs within 2% to 3% degradation of the MBGD results for the respective batch size. Overall, the *rank-seq* results are similar with the *rank-sum* ones at the equivalent rank. This is likely due to the fact that there is minimal overlap between the ranks for this additive decomposition method (Lee and Seung, 2000).

### Comparison Between the Algorithms

**FIGURE 10:**
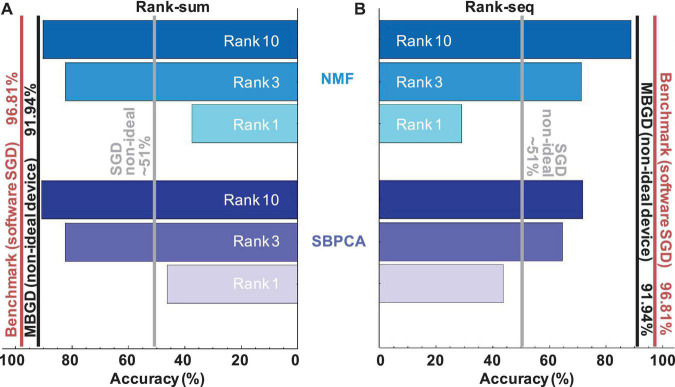
Comparison of streaming batch PCA and NMF results for rank-sum and rank-seq for a multi-layer perceptron with non-ideal memristive weights at different ranks and batch sizes. The results show that the streaming batch PCA using rank-sum slightly outperforms NMF rank-sum and can approximate very well the MBGD results at high ranks. By comparison, NMF rank-seq significantly outperforms streaming batch PCA rank-seq for higher ranks (e.g., 3 and 10) and can approximate well the MBGD results. For all these experiments, the rounding method is stochastic, batch size = 4096 and block size = 32.

The streaming batch PCA shows the most efficient compression of the batch gradient information. It obtains better accuracies than NMF for all ranks and batch sizes when the *rank-sum* transfer method is used. Streaming batch PCA *rank-sum* for rank 10 has an accuracy equivalent to MBGD ≈ 91.5% for batch size 4096. This result is around 5 percentage points lower than the traditional 64-bit floating-point algorithmic implementation for MNIST training at batch size 1 (SGD) which is the target / benchmark result for this work. This result, summarized in Figure 10 and Table 2, shows that decomposition methods in conjunction with large batch size MBGD training can overcome memristive synaptic device non-idealities and achieve close to software-equivalent accuracies.

**TABLE 2:** Summary of the best results for different ranks, batch sizes and truncation methods for streaming batch PCA vs. NMF.

		Streaming Batch PCA		NMF	
Data type		Rank-sum accuracy (%)	Rank-seq accuracy (%)	Rank-sum accuracy (%)	Rank-seq accuracy (%)
SGD		51.04			
MBGD 128		86.49			
MBGD 4096		91.94			
Batchsize 128 Block 32	Rank1	58.60	58.08	24.15	31.02
	Rank3	80.04	61.97	71.87	70.06
	Rank10	87.30	64.76	83.26	82.03
Batchsize 4096 Block 32	Rank1	46.37	43.71	37.38	29.09
	Rank3	82.33	64.71	82.15	71.27
	Rank10	90.65	71.78	89.93	88.87
Batchsize 4096 Block 128	Rank1	37.49	51.84	25.26	26.02
	Rank3	81.28	63.80	81.44	76.1
	Rank10	90.78	69.52	90.12	89.39
Batchsize 4096 Block 512	Rank1	40.30	52.37	18.63	27.37
	Rank3	85.31	63.19	76.83	76.10
	Rank10	91.03	71.41	90.33	88.17
	Rank1	45.29	48.37	27.48	25.69
Batchsize 4096 Block 1024	Rank3	84.40	65.81	75.77	77.79
	Rank10	91.48	72.10	90.28	89.08

*Realistic device model used for all these results.*

However, streaming batch PCA has its challenges. The main problem is that it operates on the eigenspace of the entire synaptic weight matrix, statistically representing the direction of largest variance, but there is no clear spatial explanation for negative numbers. Therefore the transfer of the gradient information into the memristor matrix by mapping the gradient data to number of pulses for the update (open loop transfer) is challenging as principal components can have positive and negative signs leading to inefficient oscillatory programming. For this reason, rank-by-rank weight transfer *rank-seq* underperforms by comparison with *rank-sum* for streaming batch PCA. It is important to point out that oscillatory behavior *per se* can be supported by resistive crossbar arrays via successive increase and decrease in conductance. The devices can be tuned with desired precision, but it might take very long trains of pulses and it is not desirable from a speed perspective when using devices with non-linearity and variability. If positive and negative updates to the weight are needed in rapid succession, the device programming becomes very inefficient. Therefore the transfer of the gradient information into the physical device matrices by mapping the gradient data to number of pulses for the update is challenging. In comparison, NMF calculates an approximate matrix factorization with separate positive and negative gradient information which causes the updates to avoid overlapping with one another.

By avoiding overlapping ranks, NMF has superior performance at high ranks by comparison with streaming batch PCA. For example, at rank 3, NMF *rank-seq* outperforms streaming batch PCA *rank-seq* by ≈ 5%. At rank 10, the gap is 17%. The best *rank-seq* accuracy is obtained by NMF rank 10 (88.87%) and it is less than 2% lower than the best *rank-sum* accuracy obtained via streaming batch PCA at rank 10 (90.65%). This means in practice that the NMF factorization produces the set of optimally efficient rank 1 update operations to training memristor neural networks.

The main drawback of applying the proposed methodology is related to accuracy. MBGD, particularly at large batch sizes, has lower accuracy than lower batch sizes (Goyal et al., 2017; Golmant et al., 2018). Furthermore, low rank decompositions of the MBGD gradient information can negatively affect accuracy when large networks and complex datasets are used for training. The results of this work show that it is possible to obtain low rank decomposition accuracies as close as 2% to 3% from the MBGD accuracies when large batch sizes are used. This slight penalty in accuracy comes at the potential advantage of large storage capacity for the network parameters. This tradeoff needs to be investigated further by taking accuracy targets, hardware overhead, network layer sizes, and other hyperparameters into consideration.

However, their full potential can only be explored on dedicated hardware co-processors. For example, the Streaming Batch PCA algorithm requires computationally intensive QR factorization (Huang et al., 2020a). The NMF algorithm requires explicit calculation of the full batch matrix to get the separate non-negative components. Optimized NMF algorithms mappable to hardware co-processors need to be developed, e.g., streaming variants (see Section 2 and Supplementary Figure 2). These limitations can be overcome in dedicated hardware accelerators, e.g., based on systolic arrays. A discussion of the hardware considerations is included in the Supplementary Material. Issues related to energy efficiency and speed need hardware models for the decomposition modules to be integrated with the existing circuit and device models as part of a comprehensive design verification framework (Hoskins et al., 2021).

### Applicability and Scale-Up Potential

In general, the proposed algorithms should be broadly applicable to any family of weights arrays in a matrix where the weights are trained by gradient descent. These simulation results highlight the potential of low-rank gradient decompositions in neural networks using memristor weights and are the first steps toward training co-processors to support the scale-up of machine learning models in such hardware. Several recent works demonstrate the applicability of memristor crossbars to recurrent and convolutional neural networks (Li et al., 2019; Wang et al., 2019; Lin et al., 2020). The same decomposition and implementation principles could be applied to fully connected recurrent layers. For a convolutional network, the fully connected layers performing the classification in a deep network can benefit from these decomposition methods. It is therefore possible to consider the application of the proposed methods to deeper, more complex networks.

For spiking neural networks, this property can prove important since gradient based methods have recently taken on renewed popularity in the training of such networks, especially through the use of surrogate gradient methods (Neftci et al., 2019). An increasingly common practice, despite the lack of biological plausibility, is to use mini-batch GPU acceleration of spiking networks to train them more rapidly (Neftci et al., 2017; Payvand et al., 2020). While researchers cite that future hardware will be able to more efficiently train using batch sizes of 1 (Stewart et al., 2020), this has also frequently been proposed as the ideal batch size for using memristor-based artificial neural networks due to the memory overhead associated with gradient data. However, as shown in this work, low batch size training leads to catastrophically poor performance and larger batch sizes are needed to improve training of non-ideal hysteretic devices.

Our approach to compress gradient based information as presented here could be an important step toward developing biologically plausible batch averaging during long term learning. The methods can be adapted to require only local neuronal information, thus leading to methods resilient to local nanodevice non-idealities. Compression algorithms similar to the ones studied here, e.g., Oja’s learning rule (Oja, 1982), were initially introduced as biologically plausible means to learn incoming data. Therefore, they could be used in a realistic way to efficiently learn surrogate gradients during the training of spiking neural networks.

## Conclusion

This paper investigated mini-batch training and gradient decomposition algorithmic methods to overcome the hardware non-idealities of memristor-based neural networks. By testing two different decomposition methods (streaming batch PCA and NMF) and two different weight transfer methods (*rank-sum* and *rank-seq*) for different memristor device models and ranks, we showed that the combination of the above methods is a feasible method for training the fully connected networks implemented with non-ideal devices via rank 1 updates. Our results indicate that stochastic rounding can overcome the loss of precision due to the quantization error from the vanishing gradient issue, which is of particular importance when it comes to the synaptic devices of low-bit precision, such as memristors. While the low-rank decomposition methods both produced accuracies close to those of full-rank MBGD, the choice of the update method was particularly significant for the gradient information transfer to the memristor matrix hardware. Overall, NMF produced a less efficient compression of the batch gradient than that of streaming batch PCA. However, we speculate that it was better for the rank-by-rank transfer to the memristor crossbar since all the gradient components were additive, thus eliminating the effect of device update hysteresis, though this needs further investigation. The *rank-seq* NMF is more in line with the physical constraints of memristor synaptic weights and may represent the optimal set of rank-1 updates that can be used to train a memristor array in an open loop fashion.

Future work will focus on expanding these results to deeper networks, including other types of layers, such as recurrent layers and applicability to spiking neural networks. In addition, other hardware-aware decomposition methods will be investigated. This methodology can be applied to neural networks implemented with other types of non-volatile memory devices such as phase change memory and flash technology. Ultimately, the goal is to test these proposed algorithms in full hardware implementations in memristor-based accelerators that demonstrate software equivalency despite device non-idealities.

## Data Availability Statement

The raw data supporting the conclusions of this article will be made available by the authors, without undue reservation.

## Author Contributions

JZ and SH developed the decomposition code modules and performed the simulations. OY helped with the execution time analysis. YG helped with the mini-batch gradient descent code. BH provided guidance and support. GA supervised the work. All authors participated in data analysis, discussed the results, and co-edited the manuscript.

## Conflict of Interest

The authors declare that the research was conducted in the absence of any commercial or financial relationships that could be construed as a potential conflict of interest.

## Publisher’s Note

All claims expressed in this article are solely those of the authors and do not necessarily represent those of their affiliated organizations, or those of the publisher, the editors and the reviewers. Any product that may be evaluated in this article, or claim that may be made by its manufacturer, is not guaranteed or endorsed by the publisher.
