# Supplementary material for: Gradient Decomposition Methods for Training Neural Networks With Non-ideal Synaptic Devices
Source: Front Neurosci. 2021 Nov 22;15:749811. doi: 10.3389/fnins.2021.749811 (PMC8645649; doi:10.3389/fnins.2021.749811)
Supplement: Supplementary file 1 [file Data_Sheet_1.pdf]

Supplementary Material

1. QR factorization

In the Streaming Batch PCA algorithm, the number of QR factorizations is determined by the batch size (B) / block size (b). This approach is used to reduce the frequencies of QR factorization, since it is a time-intensive computation. For the batch size 4096, we investigated the effectiveness on performance for different block sizes equal to 32, 128, 512, and 1024. As it can be noticed from **Figure S1A**, the accuracies for rank 3 and rank 10 are virtually unaffected by the chosen block size and the different QR-factorizations frequencies. These results are independent on the transfer method. **Figure S1B** shows the execution time from the backward pass with the same parameters. As the block size increases, the execution time decreases because the number of the QR factorizations per batch decreases.

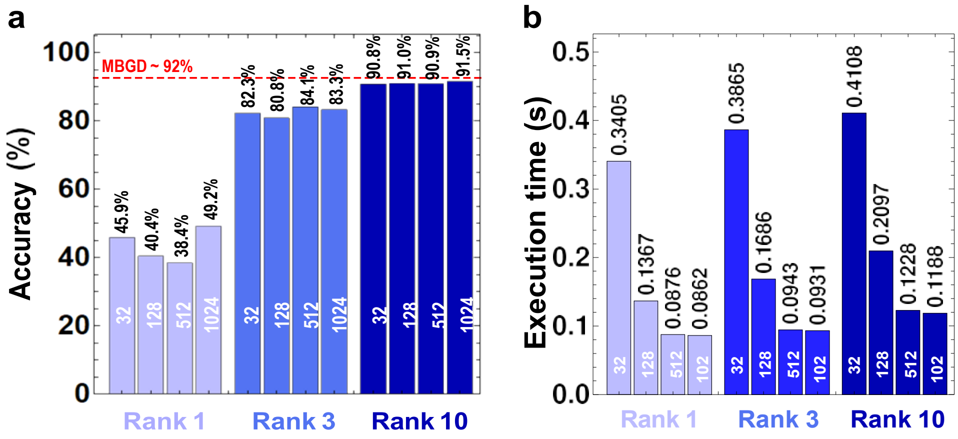

Supplementary Figure 1. Accuracy and execution time for different block sizes applied to the rank-sum streaming batch PCA results. **(A)** The accuracy results virtually unaffected by the chosen block size and the different QR decomposition frequencies. **(B)** The execution time results affected by the chosen block size and the different QR decomposition frequencies. For all these experiments, the rounding method is stochastic and batch size = 4096.

2. Towards hardware implementation

While in this work, the decomposition methods are implemented at the algorithmic level and executed on a CPU, it is possible to implement this kind of data compression efficiently in hardware by using systolic arrays composed of traditional digital circuits. Such decomposers have the advantage that the CMOS circuitry that controls the systolic array requires less short-term memory e.g. SRAM and DRAM (kilobytes to megabytes) in comparison with the long-term memristor array used for the network training and weight storage (gigabytes to terabytes). Systolic arrays have been shown to efficiently perform vector matrix multiplication (McCanny and McWhirter, 1983; Asgari et al., 2019) and various decompositions in hardware (Ahmedsaid et al., 2003b; Ahmedsaid et al., 2003a; Maltsev et al., 2006; Wang and Leeser, 2009).

Using systolic decomposers, gradient update data can be efficiently transmitted at the hardware level in compressed form into long-term memories using parallel rank-1 operations. However, this memory-aware approach requires algorithms that operate non-iteratively on streams of incoming data. Our previously proposed streaming batch PCA takes into consideration these hardware constraints and maps well to systolic decomposers (Adam et al., 2020; Daniels et al., 2021), although it has limited *rankseq* accuracy due to oscillatory behavior. This work shows that the NMF algorithm promises better *rankseq* accuracy, but optimized streaming versions need to be developed in the future. Preliminary work shows that streaming NMF, where the number of Hals iterations (p) is reduced from 200 to 1 can still achieve similar accuracy with non-streaming NMF at large batch sizes (**Figure S2a** and **b)**. Batch sizes below 128 show a convergence issue due to exploding gradients, causing the accuracy to collapse after a few epochs and the learning rate window to shrink (**Figure S2c** and **d)**. Future work will optimize the algorithmic implementation for streaming batch NMF, the systolic models for streaming batch PCA and NMF algorithms as well as determine its ASIC hardware overhead.

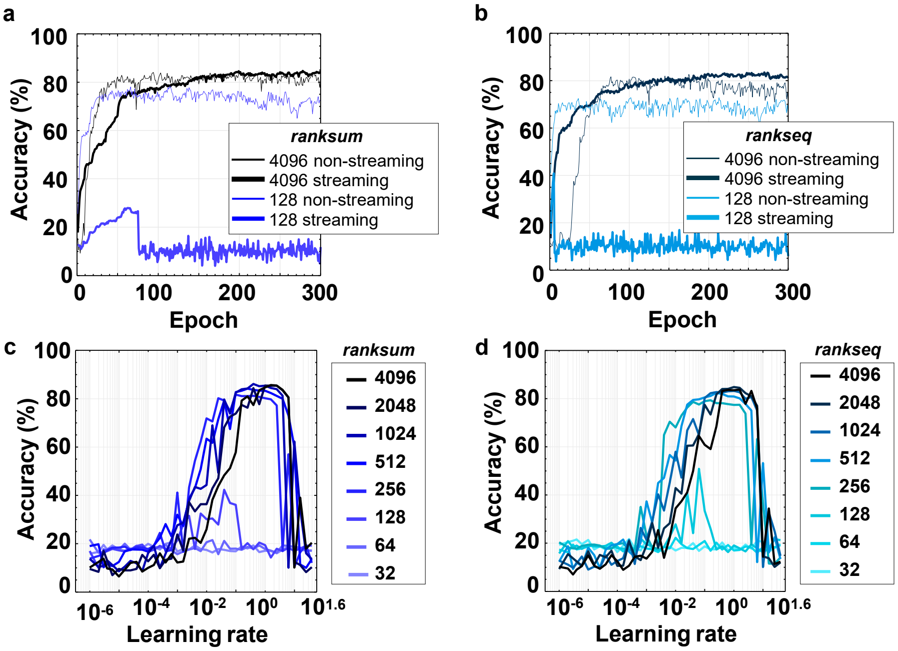

Supplementary Figure 2 Results for streaming NMF with realistic memristor devices. Comparative convergence curves between non-streaming and streaming NMF algorithm at large (4096) and small batch size (128) for **(A)** *ranksum* and **(B)** *rankseq* showing both similar accuracies at higher batch sizes; **(C)** Learning rate windows for *ranksum* and **(D)** *rankseq* for different batch sizes, showing convergence collapse at small batch sizes. MNIST and stochastic rounding used.

REFERENCES

Ahmedsaid, A., Amira, A., and Bouridane, A. (2003). Efficient systolic array for singular value and eigenvalue decomposition, in: *2003 46th Midwest Symposium on Circuits and Systems*: IEEE), 835-838.

Ahmedsaid, A., Amira, A., and Bouridane, A. (2003). Improved SVD systolic array and implementation on FPGA, in: *Proceedings. 2003 IEEE International Conference on Field-Programmable Technology (FPT)(IEEE Cat. No. 03EX798)*: IEEE), 35-42.

Asgari, B., Hadidi, R., Kim, H., and Yalamanchili, S. (2019). Eridanus: Efficiently running inference of dnns using systolic arrays. *IEEE Micro* 39(5), 46-54.

Adam, G. C., Hoskins, B.D., and Lueker-Boden, M. (2020). EigenArch: A Low Rank Hardware Machine Learning Accelerator, *DARPA Electronics Resurgence Initiative (ERI) Summit*.

Daniels, M. W., Hoskins, B.D., Madhavan, A., Yousuf, O., Adam, G., Branstad, M., Hoang, T., Madsen, R., Lueker-Boden, M., McClelland, J., and Stiles, M. D. (2021). Quasisystolic arrays for pipelined and resource-efficient neural network training, Sigma Xi: NIST.

Maltsev, A., Pestretsov, V., Maslennikov, R., and Khoryaev, A. (2006). Triangular systolic array with reduced latency for QR-decomposition of complex matrices, in: *2006 IEEE International Symposium on Circuits and Systems*: IEEE), 4 pp.

McCanny, J., and McWhirter, J. (1983). Bit-level systolic array circuit for matrix vector multiplication, in: *IEEE Proceedings G (Electronic Circuits and Systems)*: IET), 125-130.

Wang, X., and Leeser, M. (2009). A truly two-dimensional systolic array FPGA implementation of QR decomposition. *ACM Transactions on Embedded Computing Systems (TECS)* 9(1), 1-17.
